# Global Sensitivity Analysis of the Advanced ORYZA-N Model with Different Rice Types and Irrigation Regimes

**DOI:** 10.3390/plants13020262

**Published:** 2024-01-17

**Authors:** Ya Gao, Chen Sun, Tiago B. Ramos, Junwei Tan, Ana R. Oliveira, Quanzhong Huang, Guanhua Huang, Xu Xu

**Affiliations:** 1 State Key Laboratory of Efficient Utilization of Agricultural Water Resources, China Agricultural University, Beijing 100083, China; yagao1995@cau.edu.cn (Y.G.); huangqzh@cau.edu.cn (Q.H.); ghuang@cau.edu.cn (G.H.); xushengwu@cau.edu.cn (X.X.); 2 Institute of Environment and Sustainable Development in Agriculture, Chinese Academy of Agricultural Sciences, Beijing 100081, China; 3 Centro de Ciência e Tecnologia do Ambiente e do Mar (MARETEC), Instituto Superior Técnico, Universidade de Lisboa, Av. Rovisco Pais nº. 1, 1049-001 Lisboa, Portugal; tiagobramos@tecnico.ulisboa.pt (T.B.R.); anaramosoliveira@tecnico.ulisboa.pt (A.R.O.)

**Keywords:** global sensitivity analysis, Extended FAST, ORYZA-N model, double-season rice, single-season

## Abstract

Identifying important parameters in crop models is critical for model application. This study conducted a sensitivity analysis of 23 selected parameters of the advanced rice model ORYZA-N using the Extended FAST method. The sensitivity analysis was applied for three rice types (single-season rice in cold regions and double-season rice (early rice and late rice) in subtropical regions) and two irrigation regimes (traditional flood irrigation (TFI) and shallow–wet irrigation (SWI)). This study analyzed the parameter sensitivity of six crop growth outputs at four developmental stages and yields. Furthermore, we compared the variation in parameter sensitivity on model outputs between TFI and SWI scenarios for single-season rice, early rice, and late rice. Results indicated that parameters RGRLMX, FRPAR, and FLV0.5 significantly affected all model outputs and varied over developmental stages. Water stress in paddy fields caused by water-saving irrigation had more pronounced effects on single-season rice than on double-season rice.

## 1. Introduction

China is among the biggest consumers of nitrogen fertilizer worldwide, and its rice fields account for around 40% of global nitrogen fertilizer use [1]. It is well-known that water and nitrogen are the two major elements that affect rice growth and yield formation in field management. Traditional family-holder agriculture usually applies excessive irrigation and fertilization management approaches for paddy fields in pursuit of high yields, which brings many environmental problems such as soil consolidation, surface source pollution, and greenhouse effects [2,3,4]. Investigating reasonable water and nitrogen management practices in rice fields by means of field trials is often time-consuming and laborious, and the accuracy of measurement results is limited by the experimental conditions [5,6]. In addition, field trials are often influenced by different hydrological year patterns and agrometeorological conditions. Crop models are proposed on the basis of agricultural processes and mathematical methods, which can effectively complement the deficiencies in field experiments [7,8,9]. In recent years, crop models have been widely used in yield prediction, agro-ecological process simulation, field experimental management, and climate change reflections [10,11]. Accordingly, the reliability of predictions is crucial for farmers, researchers, and policy makers.

Crop models (generally developed based on multi-processes) have complicated and nonlinear properties involving numerous parameters in various modules (i.e., soil water, crop growth, management practices, meteorological environment, etc.). Many parameters and their value ranges vary greatly for crop varieties, soil environments, and agronomy management, thus limiting their direct determination through experiments [12,13]. Furthermore, the modules and parameters in crop models are numerous and exist in equifinality. It then becomes difficult to obtain suitable parameter estimation results by a trial-and-error method. Meanwhile, the parameter calibration results are easily influenced by the testers’ subjectivity, resulting in great uncertainty [14].

Parameter sensitivity analysis is usually applied to find out essential parameters in a crop model and greatly reduce the dimensionality of parameter space, which is significantly important for the improvement of model structure and parameter estimation efficiency. Sensitivity analysis (SA) is an effective approach to compute the impacts of unknown factors (parameters or driving variables) on the model’s output variables in a qualitative or quantitative manner [15,16,17]. SA methods are generally classified into two types: local and global SA techniques. Global SA is widely used in crop models, and the most representative methods are the Morris screening method, FAST (Fourier Amplitude Sensitivity Test), Sobol, and Extended FAST (EFAST) based on the variance method [18,19,20,21]. Extended FAST is an outstanding global SA method that has been adapted to many crop models, such as WOFOST [7], EPIC [22], AquaCrop [5], WARM [10], and APSIM [23].

Model parameters usually have different sensitivities under different agro-meteorological conditions [8,24]. Previous studies show that key parameters may be different between single-season rice and double-season rice (i.e., early rice and late rice) in cold regions and tropical/subtropical regions due to their various growth environments (e.g., cultivars, temperature, light, and radiation conditions) [11,25,26]. In the process of model parameter calibration, it is necessary to analyze the parameter sensitivity to different crop growth environments in order to produce more informative results.

The ORYZA model, which is extensively used worldwide, has demonstrated its reliability in simulating rice growth and paddy water balance across various experimental sites and cultivars [11,26,27,28]. Due to insufficient evidence and uncertainties in field experiments, the coupled effects of water and nitrogen in paddy fields have remained controversial over recent years [15,29]. In addition, the sensitivity of nitrogen-related parameters in crop models is still unknown. However, the validation of water and nitrogen coupling effects in paddy rice fields is crucial for simulating rice growth and development, formulating reasonable water and nitrogen management measures, and improving water and nitrogen use efficiency in paddy fields.

The ORYZA-N model (proposed by Gao et al. (2023) [30]) is an advanced soil–water–nitrogen–plant coupling model for paddy rice fields, which particularly strengthens the simulation capability of nitrogen balance and proves its accuracy and reliability. The stability and universal applicability of this advanced model still deserve to be further explored, especially for its use in different climatic zones [30,31,32]. In this study, we applied the Simlab software (ver. 2.2) to implement the Extended FAST method and achieve the following objectives: (1) to improve the application of ORYZA-N model to rice in typical climate regions (i.e., the single-season rice in cold regions and the double-season rice in subtropical regions); (2) based on the fully coupled water and nitrogen characteristics of the ORYZA-N model, to clarify the sensitive parameters on biomass and nitrogen related outputs at different development stages; and (3) to test the model stability and applicability in rice growth, yield formation, and nitrogen uptake simulation in typical climate regions. This study aims to simplify the input parameterization process for the ORYZA-N model and provide important calibration and evaluation suggestions for future model applications to rice cultivars in different climate regions.

## 2. Results and Discussion

### 2.1. Sensitivity Indices of Parameters for Model Crop-Related Outputs at Different Development Stages

#### 2.1.1. Basic Vegetative Phase

**Figure 1:**
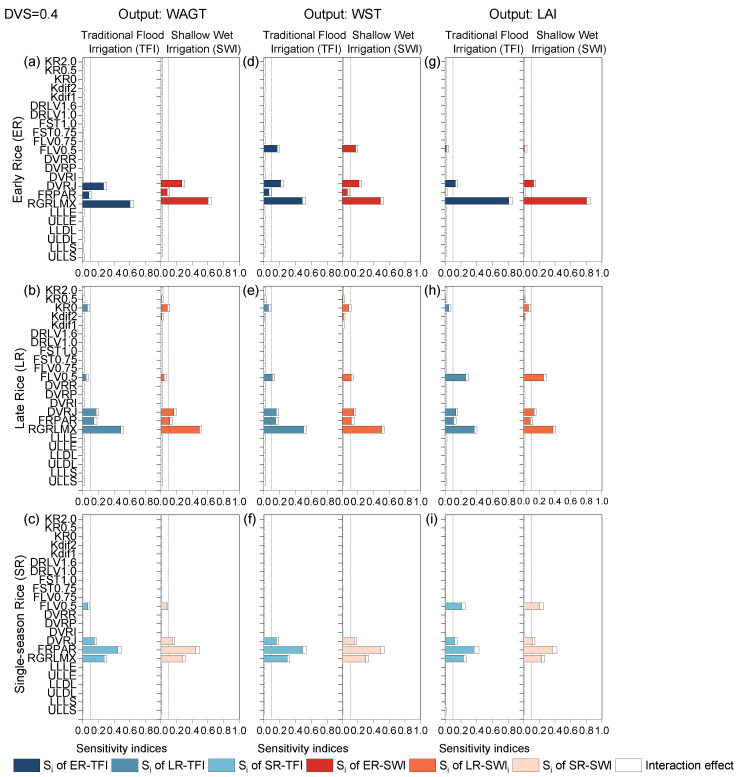
First-order sensitivity indices (Si) and interaction effects of the ORYZA-N model at DVS = 0.4: crop-related outputs WAGT (**a**–**c**), WST (**d**–**f**), and LAI (**g**–**i**) for early rice, late rice, and single-season rice under traditional flood irrigation and shallow–wet irrigation conditions.

The sensitivity indices of 23 model parameters for crop-related outputs WAGT (a–c), WST (d–f), and LAI (g–i) at the end of the basic vegetative phase (DVS = 0.4) for early rice, late rice, and single-season rice under TFI and SWI regime were presented in Figure 1. At this stage, the parameter sensitivities of the same output variables for each rice type hardly differed under different irrigation regimes. However, the sensitivity effects of the 23 parameters varied a lot for different output variables of different rice types. The SA results indicated that the developmental rate in the juvenile phase (DVRJ), the sensitivity effects of the photosynthetically active sunlight energy radiation fraction parameter (FRPAR), and the maximum relative growth rate of leaf area (RGRLMX) were the most sensitive parameters affecting almost all crop-related outputs, with the exception of LAI of early rice, which was insensitive to the FRPAR parameter. The phenological development rate determines the rate at which the crop utilizes the average daily ambient temperature and photoperiod to generate assimilates [26]; thus, the parameter DVRJ was one of the most sensitive parameters at the beginning of rice growth. Due to the variation in early growth ambient temperatures, with single-season rice having the longest growth period, followed by early rice and late rice, the effect of the radiation coefficient FRPAR and its accumulation during the growing period could not be neglected for each rice type.

RGRLMX was the most sensitive parameter for early rice and late rice, but for single-season rice, it became the second most sensitive parameter, replaced by FRPAR at the top. FLV0.5 (at the DVS = 0.5, the fraction of shoot dry matter allocated to leaves) was found to be a sensitive parameter for WST in early and late rice, and LAI in single-season rice. Its effect on WAGT was negligible. For the output variable LAI, the sensitivity results were more similar for late rice and single-season rice, although they had different values for the SA results. However, there were significant differences between early rice and late rice, with LAI for early rice being sensitive only to DVRJ and RGRLMX. Generally, the results of this phenological phase were consistent with Tan et al. (2016) [8]. Due to the leaf morphology, higher latitude, and lower plant density [33,34,35], single-season rice in cold regions was more sensitive to FRPAR than double-season rice in subtropical regions, with the total-order sensitivity indices reaching over 0.4. It should be noted that in this model, the allocation coefficients of organs between two adjacent developmental stages were varied linearly. The sensitivity results of RGRLMX on LAI, WAGT, and WST were consistent with the results drawn from the ORYZA (v3) model [36], while results for other crop parameters were inconsistent. This was due to the different model structures of ORYZA-N and ORYZA (v3), with the former model providing a comprehensive improvement of the nitrogen module and other modules.

#### 2.1.2. Photoperiod-Sensitive Phase

**Figure 2:**
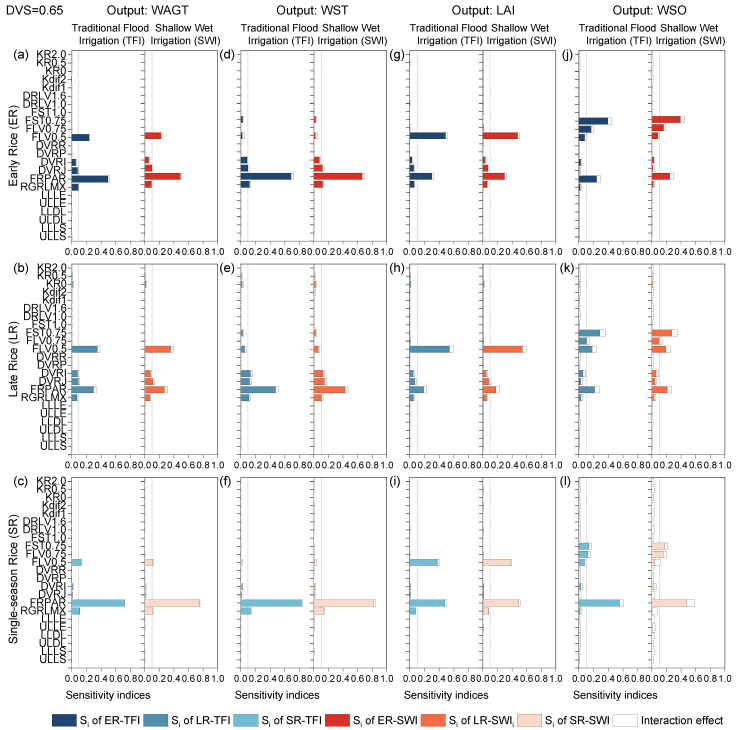
First-order sensitivity indices (Si) and interaction effects of the ORYZA-N model at DVS = 0.65: crop-related outputs WAGT (**a**–**c**), WST (**d**–**f**), LAI (**g**–**i**), and WSO (**j**–**l**) for early rice, late rice, and single-season rice under traditional flood irrigation and shallow–wet irrigation conditions.

At this stage, the biomass of panicles (WSO) began to appear. The results of SA for TFI and SWI were not significantly different for various rice varieties (Figure 2). Output results for early rice and late rice were similar for all biomass components (i.e., WAGT, WST, LAI, and WSO), and all outputs were sensitive to FRPAR. Some other outputs had inconsistent sensitivity results. For example, WAGT, LAI, and WSO were sensitive to FLV0.5, but WST was insensitive; WAGT and WST were sensitive to the phenological development rate parameter during the juvenile phase and photoperiod-sensitive phase (i.e., DVRJ and DVRI) and RGRLMX, but their effects were negligible for LAI and WSO. FST0.75 and FLV0.75 (the coefficients of shoot dry matter allocated to stems and leaves at the DVS = 0.75) only influenced WSO because the fraction of dry matter allocated to the panicles after panicle formation could be obtained by minus the portion of leaves and stems from 1.

For single-season rice, FRPAR was the most sensitive parameter affecting all crop-related outputs, ranging from 0.43 to 0.54, because of the higher latitude and light radiation. FLV0.5 was the second most sensitive parameter to LAI and WAGT, the fourth most sensitive to WSO, and insensitive to WST. WSO of single rice was also influenced by FST0.75 and FLV0.75, agreeing with WSO of double-season rice. The sensitivity observed in all crop-related outputs to RGRLMX and FLV0.5 agreed with Tan et al. (2020) [36] and Yu et al. (2023) [31], while for other parameters, results were inconsistent due to the same reasoning as in the former phase. It was worth noting that the radiation parameter (FRPAR) became the most sensitive parameter, affecting all outputs from this stage. Radiation had a positive effect on rice growth and development, and high solar radiation interception by the rice canopy was closely related to high biomass production and grain yield [37,38]. In agreement with Tu et al. (2022) [39], radiation was one of the most influential factors in rice growth.

#### 2.1.3. Panicle-Formation Phase

**Figure 3:**
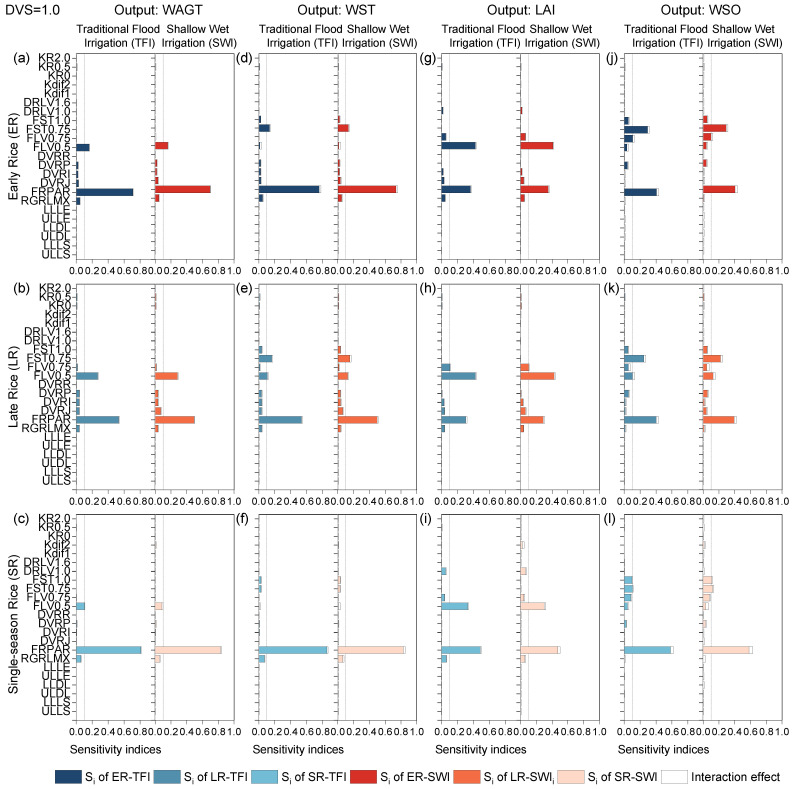
First-order sensitivity indices (Si) and interaction effects of the ORYZA-N model at DVS = 1.0: crop-related outputs WAGT (**a**–**c**), WST (**d**–**f**), LAI (**g**–**i**), and WSO (**j**–**l**) for early rice, late rice, and single-season rice under traditional flood irrigation and shallow–wet irrigation conditions.

At the panicle-formation phase, different irrigation regimes slightly altered the sensitivity values of some parameters, such as DVRJ on all outputs of early and late rice, but did not significantly affect the determination of parameter sensitivity levels (Figure 3). FRPAR was the most sensitive parameter affecting almost every crop output, except LAI for double-season rice, which was second to FLV0.5. Most outputs were sensitive to FLV0.5, except WST and WSO of single-season rice and early rice (Figure 3). Because single-season rice and early rice had similar environmental growth temperatures during the early period, they exhibited similar sensitivity results.

Additionally, the difference in the level of parameter sensitivity resulting from the types of rice was present only in those parameters that had an equal effect on the variation of output, which showed a poor rule of variation. With the exception of the radiation parameters, WSO was strongly influenced by the dry matter partition coefficients of the organs within the stage. Since shallow–wet irrigation did not cause severe water stress, the sensitivity results of the different irrigation regimes were not significantly different until the current phase, and the crop-related output sensitivity was almost the same under both irrigation regimes. The sensitivity results were consistent with the calibration and validation procedures in our previous papers [11,30].

#### 2.1.4. Grain-Filling Phase

**Figure 4:**
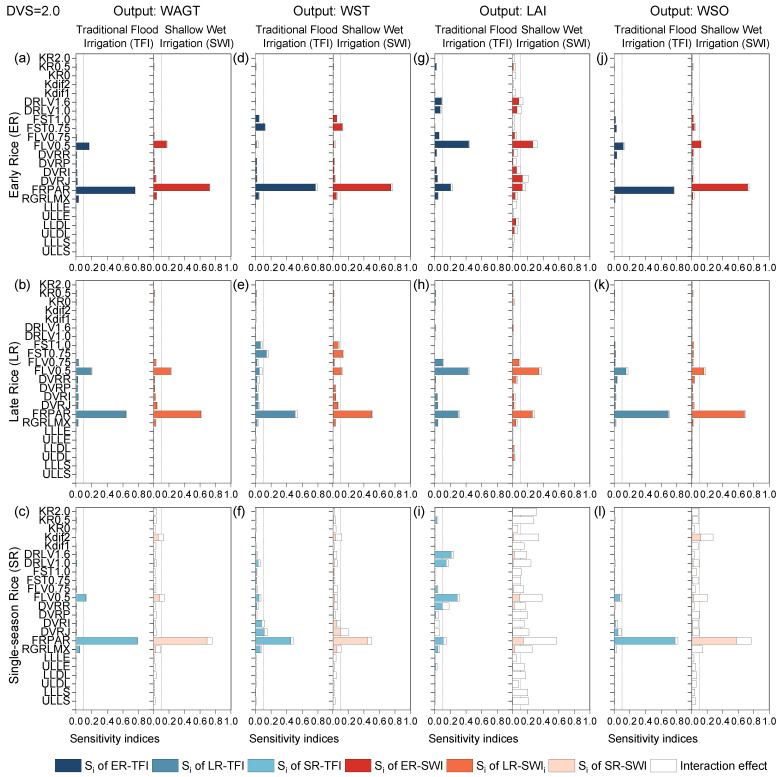
First-order sensitivity indices (Si) and interaction effects of the ORYZA-N model at DVS = 2.0: crop-related outputs WAGT (**a**–**c**), WST (**d**–**f**), LAI (**g**–**i**), and WSO (**j**–**l**) for early rice, late rice, and single-season rice under traditional flood irrigation and shallow–wet irrigation conditions.

All outputs were sensitive to parameter DVRJ during the vegetative growth period (before panicles form) but not sensitive during the reproductive growth period (after panicles form). Compared with previous stages, the effect of rice type on the sensitivity results for each section of biomass hardly changed and was mainly concentrated on single-season and double-season rice, whereas the sensitivity levels varied a little for double-season rice itself (Figure 4). The difference in sensitivity between single-season and double-season rice should be attributed to the varying ambient temperatures and climates of the planting areas [23]. FRPAR and FLV0.5 were the most sensitive and second most sensitive parameters influencing all outputs, with WST also affected by FST0.75. LAI differed from other outputs for both single-season and double-season rice, and was sensitive to multiple parameters, such as FRPAR, FLV0.5, DVRJ, the fraction of leaf death coefficient at the DVS = 1.0 and 1.6 (DRLV1.0 and DRLV1.6), the phenological development rate in reproductive phase DVRR, and FLV0.75 (Figure 4g–i). LAI at rice maturity was sensitive to leaf death rate (DRLV1.0, DRLV1.6) due to the fact that LAI was closely related to leaf biomass and decreased when the senescence death rate of leaves accelerated. This was consistent with the findings of Liu et al. (2019) [23]. At the same time, leaves that could photosynthesize were reduced, and assimilated dry matter mass was consequently decreased so that the panicle biomass was also affected. The sensitivity results were consistent with Liu et al. (2019) [23] but differed from Tan et al. (2016 and 2020) [8,36], where the effects of DRLV1.0 and DRLV1.6 were null. This was due to the difference in temperature and climate of the growing environment.

The effects of different irrigation regimes (i.e., TFI and SWI) on the sensitivity of model output variables were mainly observed in all model outputs for single-season rice. For double-season rice, the irrigation regime mainly affected LAI. LAI and WSO for single-season rice were sensitive to multiple parameters, and the interaction among the parameters had a significant effect on parameter sensitivity, indicating that for single-season rice, SWI could cause a degree of water stress by rapidly drying out the ponded water layer during rice maturity period, which was detrimental to leaf growth and rice yield formation (Figure 4i). In addition, the interaction effect between parameters increased, especially for LAI and WSO in single-season rice (Figure 4i,l). Since the ponded water layer in cold regions has a thermal insulation effect on crops, single-season rice in cold regions was more sensitive to irrigation regimes (i.e., the water stress caused by irrigation amount) than double-season rice [11,40]. The interactions among parameters were stronger for single-season rice under water-saving irrigation conditions (SWI) since the crop experienced greater water and nitrogen stress, similar to other modeling results of Liang et al. (2017) [15] and Wang et al. (2021) [41].

### 2.2. Sensitivity Indices of Parameters for Model Nitrogen-Related Outputs at Different Development Stages

#### 2.2.1. Basic Vegetative Phase

**Figure 5:**
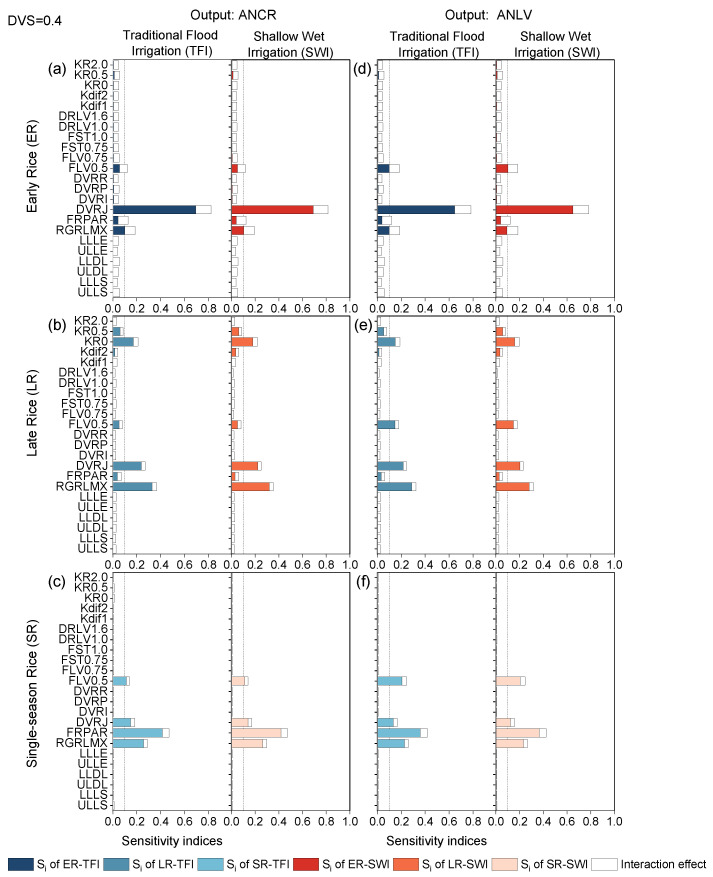
First-order sensitivity indices (Si) and interaction effects of the ORYZA-N model at DVS = 0.4: nitrogen-related outputs ANCR (**a**–**c**) and ANLV (**d**–**f**) for early rice, late rice, and single-season rice under traditional flood irrigation and shallow–wet irrigation conditions.

At the basic vegetative stage, the different irrigation regimes barely affected the sensitivity levels of the nitrogen-related outputs ANCR and ANLV (Figure 5). The sensitivity results between ANCR and ANLV for different rice types were also in good agreement. Nevertheless, there was no pattern in the sensitivity of these outputs to the selected parameters. For the same output, the impact parameters under different climatic conditions were roughly the same, but the order tended to be different, which was consistent with the findings of Liu et al. (2019) [23]. For example, DVRJ was the most sensitive parameter affecting ANCR and ANLV in early rice, the second sensitive parameter in late rice, as well as the third/fourth sensitive parameter in single-season rice. RGRLMX was the most sensitive parameter for late rice, while FRPAR had the greatest effect on single-season rice (Figure 5). FLV0.5 should also be considered when calibrating parameter values for different rice types. Note that ANCR and ANLV of late rice were sensitive to the coefficient of solute uptake from soil by rice roots at DVS = 0 and DVS = 0.5 (KR0 and KR0.5). This was because nitrogen content within rice affects photosynthetic assimilation capacity, and a decrease in nitrogen content was accompanied by a decline in the ability to capture light [26,42]. The outputs ANCR and ANLV were more sensitive to KR0 and KR0.5 in late rice, where the ambient temperature was suitable during the seedling stage of rice to meet the demands of rapid rice growth. In general, the sensitivity levels of these influential parameters were the same, although some sensitivity indices varied slightly.

#### 2.2.2. Photoperiod-Sensitive Phase

**Figure 6:**
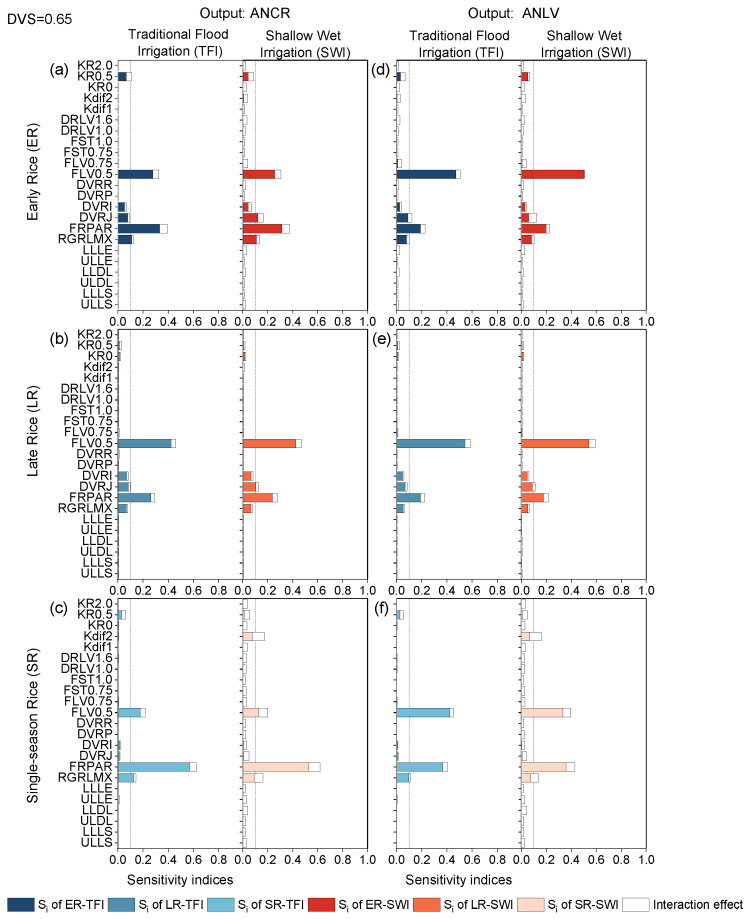
First-order sensitivity indices (Si) and interaction effects of the ORYZA-N model at DVS = 0.65: nitrogen-related outputs ANCR (**a**–**c**) and ANLV (**d**–**f**) for early rice, late rice, and single-season rice under traditional flood irrigation and shallow–wet irrigation conditions.

The growth stage of DVS = 0.4–0.65 was the rapid growth period of rice stems and leaves development, and the whole parameter sensitivity was different from the previous stage, but the parameters that had impacts on the outputs were approximately the same, namely FRPAR, FLV0.5, RGRLMX, DVRI, and DVRJ (Figure 6). At this stage, for the selected model outputs ANCR and ANLV, the sensitivity results were relatively similar for early rice and late rice, but the parameter values for the SA results were different (Figure 6a–d). For example, for double-season rice, FRPAR was the most sensitive parameter affecting the ANCR of early rice, while FLV0.5 was the most sensitive parameter for other outputs. The SA values of ANCR and ANLV for single-season rice were also different (Figure 6c,f).

In addition, the sensitivity results were approximately the same for different irrigation regimes, while the sensitivity parameters differed for different rice types (mainly between single-season and double-season rice). The nitrogen-related outputs (ANCR and ANLV) of single-season rice were sensitive to FLV0.5, FRPAR, and RGRLMX, which was consistent with previous ORYZA-N model simulations using field trial data [11]. It was noteworthy that ANCR and ANLV of single-season rice under the SWI condition were also sensitive to the solute (i.e., NH_4_-N and NO_3_-N) diffusion coefficient below the soil surface (Kdif2). Nitrogen uptake and utilization in rice under water-saving irrigation was higher than that under flood irrigation [43], and thus, the influence of nitrogen diffusion on crop N uptake was also expanded.

#### 2.2.3. Panicle-Formation Phase

**Figure 7:**
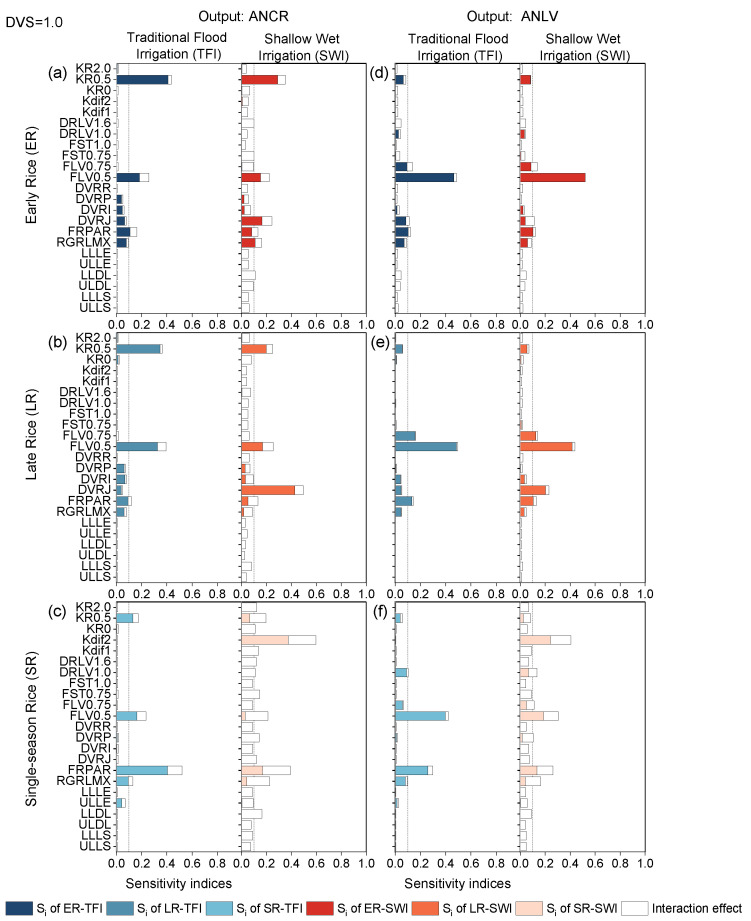
First-order sensitivity indices (Si) and interaction effects of the ORYZA-N model at DVS = 1.0: nitrogen-related outputs ANCR (**a**–**c**) and ANLV (**d**–**f**) for early rice, late rice, and single-season rice under traditional flood irrigation and shallow–wet irrigation conditions.

During panicle formation, the sensitivity of double-season rice under different irrigation regimes was affected by paddy water conditions, whereas the sensitivity for single-season rice was irregular. Under TFI conditions, ANCR was mainly affected by the variation of KR0.5, FLV0.5, and FRPAR, although the three parameters had different magnitudes of sensitivity. KR0.5 was the most sensitive parameter of ANCR for early rice, the second sensitive parameter for late rice, and the third sensitive parameter for single-season rice (Figure 7a–c).

ANLV of double-season rice was influenced by FLV0.5, FST0.75, and FRPAR, which should not be neglected, and the sensitivity indices of the most sensitive parameter, FLV0.5, were about 0.5. While the output of single-season rice mainly depended on the distribution rate, death rate, and photosynthetic effective utilization of leaves, the sensitivity results of ANLV were similar to LAI. Due to the difference in climate types between double- and single-season rice growing regions, the effect of paddy water conditions (caused by different irrigation regimes) was more noticeable for single-season rice.

The sensitivity pattern difference between ANCR and ANLV is not limited to single-season rice. Sensitivity patterns changed across all rice types. For early rice, the major flip occurred at parameters KR05, FLV05, and FLV075; meanwhile, the changed parameters were KR05, KDif2, FLV05, and FRPAR for single-season rice. SWI had a large effect on the model nitrogen outputs sensitivity to parameters, especially on single-season rice, and comparison of Figure 7c,f showed that ANCR and ANLV of single-season rice at SWI were affected by a combination of multiple parameters, the most influential of which were Kdif2 and FLV0.5, respectively. Similar to the previous stage, due to the water stress caused by SWI during this period, when conducting model calibration and validation under controlled irrigation regimes, the effect of the solute diffusion below the soil surface coefficient Kdif2 on ANCR must be taken into consideration.

#### 2.2.4. Grain-Filling Phase

**Figure 8:**
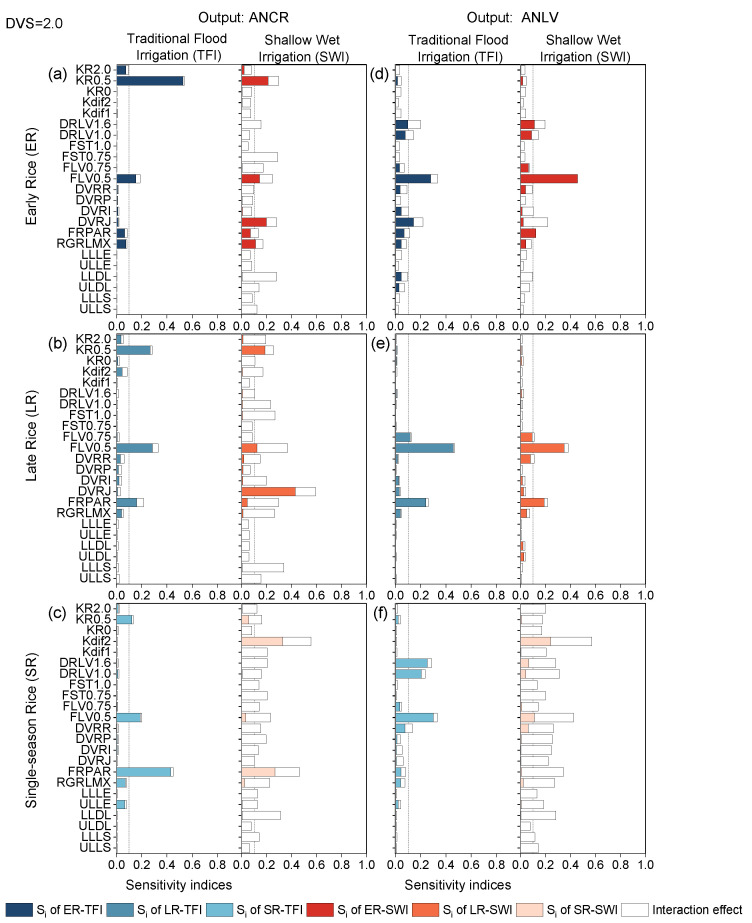
First-order sensitivity indices (Si) and interaction effects of the ORYZA-N model at DVS = 2.0. Nitrogen-related outputs ANCR (**a**–**c**) and ANLV (**d**–**f**) for early rice, late rice, and single-season rice under traditional flood irrigation and shallow–wet irrigation conditions.

Different irrigation regimes had significant effects on N-related outputs for different rice types. At this phase, the sensitivity parameters, sensitivity change patterns, and sensitivity change magnitude were quite different on ANCR and ANLV of each rice type, compared with the previous periods. ANCR was sensitive to KR0.5, FLV0.5, and FRPAR in both single- and double-season rice, and the sensitivity values of the SA results were different (Figure 8). For example, KR0.5 was the most sensitive parameter for early rice (*ST_i_* > 0.5), the second most sensitive parameter for late rice (*ST_i_* was about 0.3), and the third most sensitive parameter for single-season rice (*ST_i_* > 0.1), which was different from the sensitivity values at the panicle-formation stage (Figure 8). Under TFI, for early rice, ANCR was influenced by KR0.5 and FLV0.5, while for late rice and single-season rice, ANCR was affected by KR0.5, FLV0.5, and FRPAR.

The interaction among parameters under SWI was obvious; the ANCR and ANLV of all rice types were affected by a combination of multiple parameters, with the main sensitive parameters of double-season rice being KR0.5, FLV0.5, DVRJ, FRPAR, and RGRLMX (Figure 8a–c). This indicated that the interaction effects of nitrogen-related outputs were greater under water stress conditions, where priority should be provided to the soil water-related parameters [44]. The sensitivity results of ANCR and ANLV for single-season rice were both mainly influenced by Kdif2 and FRPAR. When focusing on the model output variable ANLV for early rice and single-season rice, the effects of DRLV1.0 and DRLV1.6 should be considered during parameter calibration. Overall, the parameter calibration and application of the nitrogen-related output should focus on the nitrogen parameters KR0.5 and Kdif2, the crop parameter FLV0.5, and the radiation parameter FRPAR.

### 2.3. Sensitivity Indices of Parameters for Yield at Maturity

**Figure 9:**
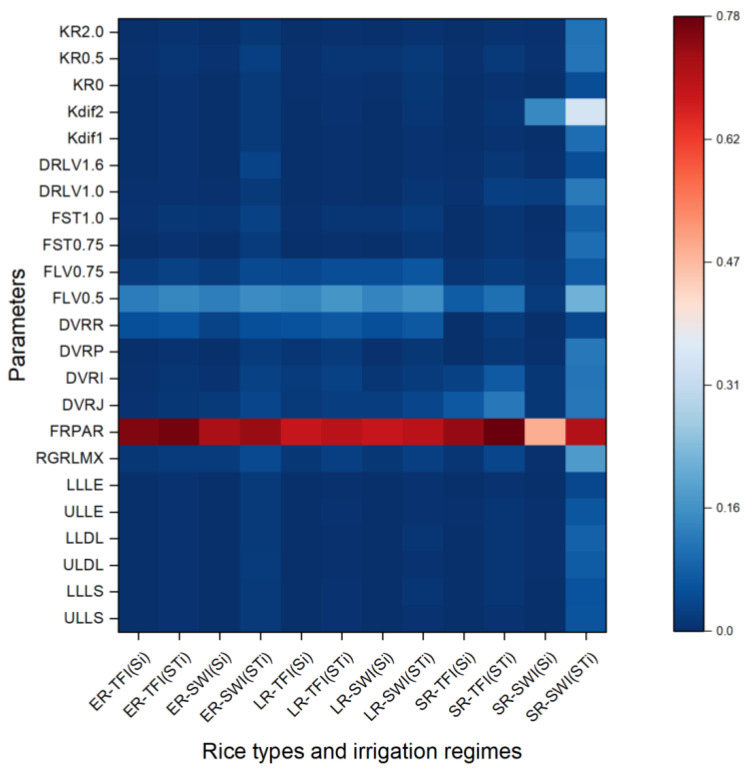
First-order (Si) and total-order sensitivity indices (STi) results of the ORYZA-N model for crop yields of early (ER), late (LR), and single-season rice (SR) under traditional flood irrigation (TFI) and shallow–wet irrigation (SWI) conditions.

Yields are usually the most focused on component of the crop models. As a result, for WRR14 (yield with 14% moisture at maturity), three rice types and two irrigation regimes were simulated, and the *S_i_* and *ST_i_* were computed for the 23 parameters (Figure 9). The results were comparable to WSO at the maturity phase (as shown in Figure 4j–l), but the differences due to rice type (i.e., growth ambient environment) were more noticeable. It was important to note that the ORYZA-N model’s WRR14 was computed using the grains number growth rate, not just the WSO at ripe.

For WRR14, FRPAR was the most important parameter, followed by FLV0.5, FLV0.75, and DVRR. For both TFI and SWI of double-season rice, the effects of all other parameters were negligible. As for single-season rice, the sensitivity of parameters under TFI was consistent with that of double-season rice, and the effect of Kdif2 on the sensitivity results was not ignorable in SWI. The developmental rate of each stage (DVRJ, DVRI, DVRP, DVRR) had a varying impact on dry biomass and nitrogen content of each organ, which also indirectly influenced the yield formation. This was consistent with the results of Yu et al. (2023) [31] and Xu et al. (2018) [38]. Since the ponded water layer of single-season rice in cold regions has the role of maintaining soil temperature in the root zone and sustaining crop growth during the growing season, controlled irrigation regimes would affect the growth and yield formation of single-season rice in cold regions at some degree, and the interaction effect among the parameters was higher than other scenarios [11,39].

## 3. Materials and Methods

In this study, we performed a parameter sensitivity analysis (SA) of the ORYZA-N model using the Extended FAST method. The ORYZA model employed the development stages (DVS) of rice growth to determine the physiological phase of the rice plant, which is characterized by the emergency of the different organs and their appearance [26]. The whole rice growth process was separated into four phenological phases: the basic vegetative phase (DVS = 0–0.4), the photoperiod-sensitive phase (DVS = 0.4–0.65), the panicle formation phase (DVS = 0.65–1.0), and the grain-filling phase (DVS = 1.0–2.0) [26]. To assess the sensitivity of model output variables to changes in the selected input parameters, we used the Simlab software [45] (https://ec.europa.eu/jrc/en/samo/simlab (accessed on 10 April 2022)) to calculate the first-order sensitivity indices and the total-order sensitivity indices of each parameter at the end of every phenological phase.

### 3.1. ORYZA-N Model

The ORYZA series model is a dynamic and mechanistic eco-physiological model that was jointly developed by International Rice Research Institution and Wageningen University. The model is designed to simulate rice growth, paddy water balance, and field management and is widely used in various regions around the world and for different cultivars [26,28,46,47]. The crop nitrogen uptake method in ORYZA2000 model is a simple bookkeeping algorithm with a daily given rate of crop nitrogen uptake, which is insufficient to quantify the nitrogen balance of the soil-crop system. To simulate and quantify the water and nitrogen balance items in paddy rice effectively, the ORYZA-N model was improved based on the ORYZA2000 version. Three new modules (i.e., a soil temperature module, an ammonium nitrogen (NH_4_-N) and nitrate nitrogen (NO_3_-N) transport module, and a soil organic carbon and nitrogen turnover module) were developed and incorporated into the ORYZA-N model. The soil water module was also improved by enhancing the calculation procedures for root water uptake and soil water drainage. ORYZA-N model was calibrated and validated using two years of field experiment data, and the results showed its reliability in the aspect of simulating the agro-hydrological processes, crop growth, and especially water–nitrogen transformation in paddy rice fields [30]. More detailed information on the ORYZA-N model can be found in Gao et al. (2023) [30].

### 3.2. Extended FAST Method

This study applied the Extended FAST method, a variance-based global SA approach, to evaluate the impact of diverse parameters from different modules on the model outputs, including dry biomass, crop nitrogen content, leaf area index (LAI), and yield, and calculate higher-order interactions among the parameters [17,48]. Extended FAST was developed on the basis of the FAST algorithm (Fourier Amplitude Sensitivity Test [49]) and the Sobol algorithm [50]. It combines the computing efficiency of the FAST method with Sobol’s ability to calculate the total effect [5,51]. The Extended FAST method used in this study has the advantages of high efficiency and accuracy and is capable of analyzing the interaction effects between parameters. Extended FAST has gained popularity in various fields, such as hydrological, ecological, and crop modeling, as well as climate change studies, owing to the advantages mentioned above [5,10,12].

The total variance *V*(*Y*) of model outputs was calculated as follows [52]:

(1) $$V\left(Y\right)=\sum_{i=1}^{n}V_{i}+\sum_{i<j\leq n}^{n}V_{ij}+\ldots +V_{12\ldots n}$$

where *V*(*Y*) indicated the total variance of model output *Y* induced by ORYZA-N model, *V_i_* ( $V_{i}=V\left[E\left(Y|x_{i}\right)\right]$ ) represented the first order for each factor, and *V_ij_* ( $V_{ij}=V\left[E\left(Y|x_{i},\;x_{j}\right)\right]-V_{i}-V_{j}$ ) to *V*_12…*n*_ represented the interactions among n factors. The variance of the conditional expectation $V\left[E\left(Y|x_{i}\right)\right]$ , also known as the main effect, was used to determine the significance of *x_i_* on the variance of *Y*, indicating the sensitivity of *Y* to *x_i_*. Extended FAST calculated two indices: the first-order sensitivity indices (*S_i_*) and total-order sensitivity indices (*ST_i_*). The *S_i_* corresponding to a single factor (*x_i_*) was calculated as follows:

(2) $$S_{i}=V_{i}/V\left(Y\right)=V\left[\left(E(Y/x_{i}\right)\right]/V\left(Y\right)$$

The *ST_i_* that correspond to a single factor (index *i*) and the interaction of more factors that involve the index *i* and at least *j* ≠ *i* from 1 to *n* was calculated by Equation (3):

(3) $$ST_{i}=S_{i}+\sum_{j\neq i}S_{ij}+\ldots +S_{1\ldots i\ldots n}=\frac{V(Y)-V_{-i}}{V(Y)}$$

Higher values denoted a greater contribution of the variable on the outcomes and vice versa.

### 3.3. Experiment and Data

Field experiment for single-season rice was carried out in 2019 at the Qing’an Irrigation Experimental Station (46°58′ N, 127°39′ E), located in the typical cold region (Songnen Plain) of northeast China. The region is characterized by a middle temperate and sub-humid continental monsoon climate, with an average annual temperature of 2.5 °C and an average annual precipitation of 635 mm. Field experimental data for double-season rice of 2012 was collected from the Jiangxi Irrigation Experiment Station in Nanchang, China (located at 28°26′ N, 115°28′ E), with a warm and humid subtropical monsoon climate. The average annual temperature and precipitation of this region were 18.1 °C and 1636 mm, respectively.

The main soil texture of both study sites is silty loam, with the main soil physical characteristics listed in Table 1. The experimental data and the phenology investigation dates used in this work were obtained in 2019 and 2012 for single-season rice in a cold region and double-season rice in a subtropical region, respectively. According to the rainfall pattern, the selected experimental years for both single- and double-season rice were wet years so that differences in the irrigation regime sensitivity caused by the climatic conditions of the experimental years (mainly rainfall) could be neglected. The dates associated with phenology, such as sowing, emergency, transplanting, panicle initiation, blooming, and maturity, were observed and recorded (Table 2). In addition, daily meteorological data, including minimum and maximum temperature, sunshine hours, relative humidity, and wind speed during the experimental years, were obtained from the nearby Tieli and Jiangxi weather stations. These data were used to calculate the reference evapotranspiration (ETo) according to the FAO Penman–Monteith method [53]. Nitrogen fertilizer application schedules for the single-season rice, early rice, and late rice during the selected experimental years are provided in Table 3 and were defined following local farmers’ practices. More detailed information regarding observations in single-season rice and double-season rice can be found in Gao et al. (2021 and 2023) [11,30] and Tan et al. (2016) [8], respectively.

### 3.4. Model Parameters and Sensitivity Analysis

In this work, 23 parameters in soil water, crop, and nitrogen modules were considered for SA (Table 4). The output state variables selected for the ORYZA-N model were dry aboveground green biomass (WAGT, kg ha^−1^), the leaf area index (LAI, m^2^ m^−2^), the dry biomass of stems (WST, kg ha^−1^) and panicles (WSO, kg ha^−1^), the nitrogen content in dry aboveground green biomass (ANCR, kg N ha^−1^) and dry biomass of leaves (ANLV, kg N ha^−1^). These outputs were analyzed at the end of the four phenological phases (i.e., DVS = 0.4, 0.65, 1.0, and 2.0).

First, the parameter values of model “warm-up” were determined based on the recommended/default parameter values of single-season rice in cold regions and double-season rice in subtropical regions from Gao et al. (2021 and 2023) [11,30] and Tan et al. (2016 and 2020) [8,36], respectively. Then, based on these parameter values and field experiment data, a rational calibration of the crop growth process was carried out for different rice types. The calibration was conducted using a trial-and-error approach, particularly aiming to provide the basic reference parameter values for SA (the comparison results are shown in Supplementary Figure S1). Both simulated and measured values showed good agreement; thus, the parameter values used in these cases could be applied as base values for SA. Next, 23 parameters related to water stress, crop growth, and nitrogen uptake/transformation were selected according to the calibration process and set to the base values obtained in the previous step. Detailed SA was finally conducted to identify the sensitive parameters for three rice types at four development stages.

Table 4 lists the base values and variance range of the selected parameters in the ORYZA-N model. The variation range of selected parameters was set as ±30% of base values as recommended by Tan et al. (2017) [29], while for four phenological parameters, the variance range of three rice types was set as ±10% because of the low ambient temperatures in the early stages of crop growth and a short suitable sowing period.

To evaluate the influence of different irrigation regimes on the parameter sensitivity results, traditional flood irrigation (TFI) and shallow–wet irrigation (SWI) regimes were considered in simulations of single-season rice, early rice, and late rice. TFI and SWI regimes were the most popular irrigation regimes in both experimental areas. The details about irrigation management at each growth stage are presented in Table 5.

In this study, the sensitivity indices *S_i_* and *ST_i_* were calculated by the Simlab (ver. 2.2) software using the Extended FAST method, as described by Tarantola [54]. Based on the knowledge of the convergence of sensitivity indices, a parameter sample size of N = 15,000 was set for the SA. This method included the following four steps: (1) Definition of parameters and their distributions. There were 6 soil water-related parameters, 12 crop-related parameters, and 5 nitrogen-related parameters. The default values and ranges are listed in Table 4. (2) Generation of sets of targeted parameters by terms of Monte Carlo distributions sampling. (3) Evaluation of model outputs for each set of selected input parameters. The ORYZA-N model was called in this process, and the results of the runs with different parameter combinations generated by Simlab were recorded through the Python code. (4) Computation of *S_i_* and *ST_i_* from the outputs resulting from variations of each input parameter produced by Simlab.

## 4. Conclusions

In this study, the Extended FAST sensitivity analysis (SA) method was used to analyze the parameter sensitivities for the ORYZA-N model in different rice growth phases. The SA was carried out for three rice types (single-season rice, early rice, and late rice) and two irrigation regimes (traditional flood irrigation (TFI) and shallow–wet irrigation (SWI)) in two typical climatic conditions (subtropical and cold temperate regions). Twenty-three parameters related to the soil-water module, crop module, and nitrogen module were selected from the ORYZA-N model to identify the sensitivity effects on six model outputs of crop growth (aboveground biomass, WAGT; dry biomass of stems and panicles, WST and WSO; leaf area index, LAI; nitrogen content in dry aboveground biomass and dry biomass of leaves, ANCR and ANLV).

In the ORYZA-N, there were only a few parameters that had significant effects on the model outputs, which could simplify the calibration procedure and provide guidance during the model application. Different rice types showed variations in the sensitivity degrees. The results showed that RGRLMX, FLV0.5, and FRPAR were the most sensitive parameters for both crop-related and nitrogen-related outputs. The sensitivity ranking of the above parameters varied among developmental stages, which should be particularly paid attention. For the three rice types, the parameter sensitivities to most outputs were similar for early rice in sub-tropical regions and single-season rice in cold regions but different from late rice. Throughout the growing periods, DVRJ, RGRLMX, and DVRI were the most sensitive parameters for biomass and nitrogen uptake during the vegetative growth period. During the reproductive growth period, FRPAR and FLV0.5 had a more prominent effect on all outputs. ANCR was sensitive to KR0.5, while ANLV was closely correlated to FLV0.75 and leaf death rate coefficients. For double-season rice (early rice and late rice), the sensitivity performance was similar under TFI and SWI regimes; meanwhile, we found that the interaction effects were stronger for water deficit conditions. For single-season rice, all outputs were sensitive to FRPAR, with stronger and more sensitive interactions than for double-season rice under water deficit conditions. For all rice types and irrigation regimes, FRPAR was the most sensitive parameter for yield (i.e., the output WRR14), followed by FLV0.5. The effect of the parameter Kdif2 on nitrogen and yield outputs could not be negligible under SWI for single-season rice.

Results also indicated that ORYZA-N has good computational and structural stability. The fully coupled water–nitrogen structure also verified the significance of the nitrogen parameter simulation process for rice modeling, which was valuable to the improvement of nitrogen simulation for other crop models. The base parameter set in this study could also complement the parameter database for rice modeling and provide useful information for the calibration and application of the advanced ORYZA-N model in different climatic regions.

## Figures and Tables

**Table 1:** Soil physical properties for different soil layers of the experimental area.

Soil Horizon (cm)	Soil Particle-Size Distribution (%)			Bulk Density (g cm^−3^)	Soil Organic Matter (%)
	Sand (2.0–0.05 mm)	Silt (0.05–0.002 mm)	Clay (<0.002 mm)		
Single-season rice in Heilongjiang province, China					
0–10	20.0	67.2	12.8	1.47	2.3
10–25	9.1	76. 9	14.0	1.48	3.3
25–40	11.3	74.9	13.8	1.53	2.5
40–80	17.8	71.7	10.5	1.29	4.0
Double-season rice in Jiangxi province, China					
0–20	8.1	70.0	21.9	1.343	2.1
20–50	11.3	67.2	21.5	1.617	1.4
50–100	6.4	71.2	23.4	1.675	0.9

**Table 2:** The observed phenology dates of the single-season rice, early rice, and late rice.

Phenology	Sowing	Transplanting	Panicle Initiation	Flowering	Maturity
Dates for single-season rice	15 April 2019	16 May 2019	10 July 2019	2 August 2019	21 September 2019
Dates for early rice	22 March 2012	21 April 2012	23 May 2012	14 June 2012	10 July 2012
Dates for late rice	21 June 2012	19 July 2012	22 August 2012	14 September 2012	19 October 2012

Note: single-season rice was observed in 2019 at Qing’an Irrigation Experiment Station, Heilongjiang province, China; early rice and late rice were observed in 2012 at Jiangxi Irrigation Experiment Station, Jiangxi province, China.

**Table 3:** Nitrogen fertilizer application schedule for the single-season rice, early rice, and late rice during the experimental years.

Treatment	Single-Season Rice (2019)		Early Rice (2012)		Late Rice (2012)	
	Date (Month–Day)	Net Nitrogen Amount (kg N ha^−1^)	Date (Month–Day)	Net Nitrogen Amount (kg N ha^−1^)	Date (Month–Day)	Net Nitrogen Amount (kg N ha^−1^)
Base fertilizer	April 27	57.6	April 19	90	July 17	90
Tillering fertilizer	May 29	33.5	April 27	54	July 29	54
Panicle fertilizer	June 15	46.4	May 26	36	August 22	36
Total		137.5		180		180

**Table 4:** Definition, base values, and variance range of the selected parameters in the ORYZA-N model.

Parameters	Definition	Unit	Default Values for Single-Season Rice (Variation Ranges)	Default Values for Early Rice (Variation Ranges)	Default Values for Late Rice (Variation Ranges)
ULLS	Upper limit of leaf rolling	kPa	74.13 (±30%)	74.13 (±30%)	74.13 (±30%)
LLLS	Lower limit of leaf rolling	kPa	794.33 (±30%)	794.33 (±30%)	794.33 (±30%)
ULDL	Upper limit of drought-induced dead leaves	kPa	630.95 (±30%)	630.95 (±30%)	630.95 (±30%)
LLDL	Lower limit of drought-induced dead leaves	kPa	1584.89 (±30%)	1584.89 (±30%)	1584.89 (±30%)
ULLE	Upper limit of leaf expansion	kPa	1.45 (±30%)	1.45 (±30%)	1.45 (±30%)
LLLE	Lower limit of leaf expansion	kPa	1404 (±30%)	1404 (±30%)	1404 (±30%)
RGRLMX	Maximum relative growth rate of leaf area	(°Cd)^−1^	0.0088 (±30%)	0.00877 (±30%)	0.00904 (±30%)
FRPAR	Fraction of photosynthetically active sunlight energy	-	0.45 (±30%)	0.5 (±30%)	0.62 (±30%)
DVRJ	Development rate during the juvenile phase	(°Cd)^−1^	0.000890 (±10%)	0.000995 (±10%)	0.001081 (±10%)
DVRI	Development rate during the photoperiod-sensitive phase	(°Cd)^−1^	0.000758 (±10%)	0.000758 (±10%)	0.000758 (±10%)
DVRP	Development rate during the panicle phase	(°Cd)^−1^	0.001055 (±10%)	0.000981 (±10%)	0.000853 (±10%)
DVRR	Development rate in reproductive phase (post-anthesis)	(°Cd)^−1^	0.002000 (±10%)	0.002139 (±10%)	0.001728 (±10%)
FLV0.5	Fraction of shoot dry matter partitioned to the leaves at DVS = 0.5	-	0.5 (±30%)	0.6 (±30%)	0.6 (±30%)
FLV0.75	Fraction of shoot dry matter partitioned to the leaves at DVS = 0.75	-	0.3 (±30%)	0.3 (±30%)	0.3 (±30%)
FST0.75	Fraction of shoot dry matter partitioned to the stems at DVS = 0.75	-	0.3 (±30%)	0.7 (±30%)	0.7 (±30%)
FST1.0	Fraction of shoot dry matter partitioned to the stems at DVS = 1.0	-	0.6 (±30%)	0.4 (±30%)	0.4 (±30%)
DRLV1.0	Fraction of leaf death coefficient at the DVS = 1.0	-	0.024 (±30%)	0.015 (±30%)	0.003 (±30%)
DRLV1.6	Fraction of leaf death coefficient at the DVS = 1.6	-	0.020 (±30%)	0.025 (±30%)	0.005 (±30%)
Kdif1	Solute diffusion coefficient of the first soil layer, which is related to solute type and conduction method	mm^2^ d^−1^	100 (±30%)	100 (±30%)	100 (±30%)
Kdif2	Solute diffusion coefficient of the next 9 soil layer	mm^2^ d^−1^	600 (±30%)	600 (±30%)	600 (±30%)
KR0.0	Relative uptake of solutes by roots from soil at DVS = 0.0	-	5 (±30%)	5 (±30%)	5 (±30%)
KR0.5	Relative uptake of solutes by roots from soil at DVS = 0.5	-	3 (±30%)	3 (±30%)	3 (±30%)
KR2.0	Relative uptake of solutes by roots from soil at DVS = 2.0	-	3 (±30%)	3 (±30%)	3 (±30%)

Note: single-season rice was observed in 2019 at Qing’an Irrigation Experiment Station, Heilongjiang province, China; early rice and late rice were observed in 2012 at Jiangxi Irrigation Experiment Station, Jiangxi province, China.

**Table 5:** Irrigation controlled criteria for two different irrigation regimes in various rice growing stages.

Irrigation Regimes	Seeding	Early Tillering	Middle Tillering	Later Tillering	Jointing and Booting	Blooming	Grouting	Yellow Ripe
	Lower Limit Criterion—Upper Limit Criterion							
Traditional flood irrigation (TFI)	30 mm– 50 mm	30 mm– 50 mm	30 mm– 50 mm	Drainage	30 mm– 50 mm	30 mm– 50 mm	30 mm– 50 mm	Naturally drying
Shallow–wet irrigation (SWI)	10 mm– 30 mm	80%*θ_s_*– 15 mm	80%*θ_s_*– 15 mm	Drainage	10 mm– 30 mm	10 mm– 30 mm	80%*θ_s_*– 15 mm	Naturally drying

Note: *θ_s_* is the volumetric saturated soil water content in root layers; the lower limit is expressed as the ponded water depth or the percentage of *θ_s_*, and the irrigation is applied when reaching the lower limit criterion; and the upper limit is the desired ponded water depth after irrigation.

## Data Availability

Data are contained within the article and Supplementary Materials.

## Supplementary Materials

The following supporting information can be downloaded at https://www.mdpi.com/2223-7747/13/2/262/s1. Figure S1: Model calibration comparisons of simulated and measured crop growth indicators for single-season rice (a,d), early rice (b,e), and late rice (c,f) involving dry aboveground green biomass (WAGT), stem biomass (WST), green leaves (WLVG) and panicles (WSO), and leaf area index (LAI). (This figure is adapted from Gao et al., 2023 [30]).
