# Supplementary figures and images for: Global Sensitivity Analysis of the Advanced ORYZA-N Model with Different Rice Types and Irrigation Regimes
Source: Plants (Basel). 2024 Jan 17;13(2):262. doi: 10.3390/plants13020262 (PMC11487419; doi:10.3390/plants13020262)

Single Rice

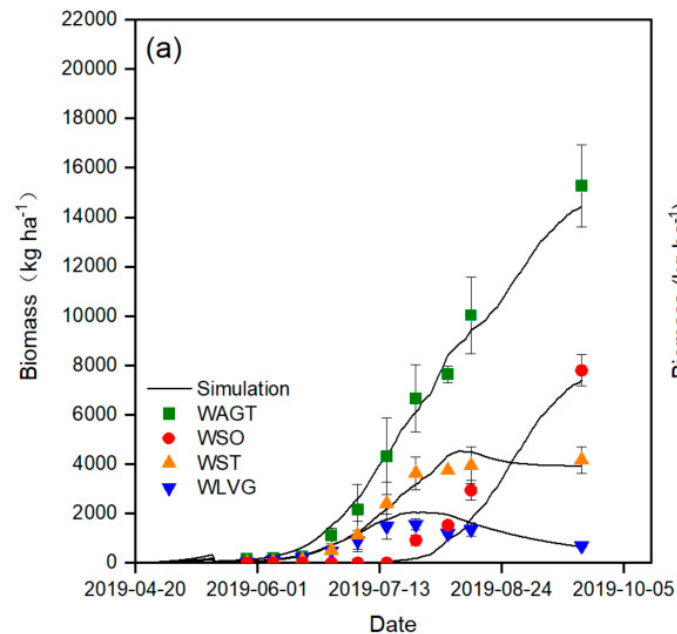

Early Rice

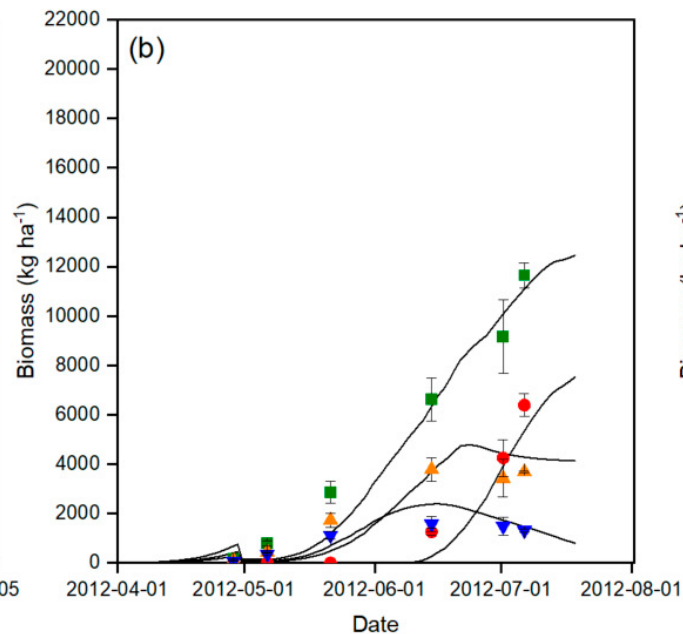

Late Rice

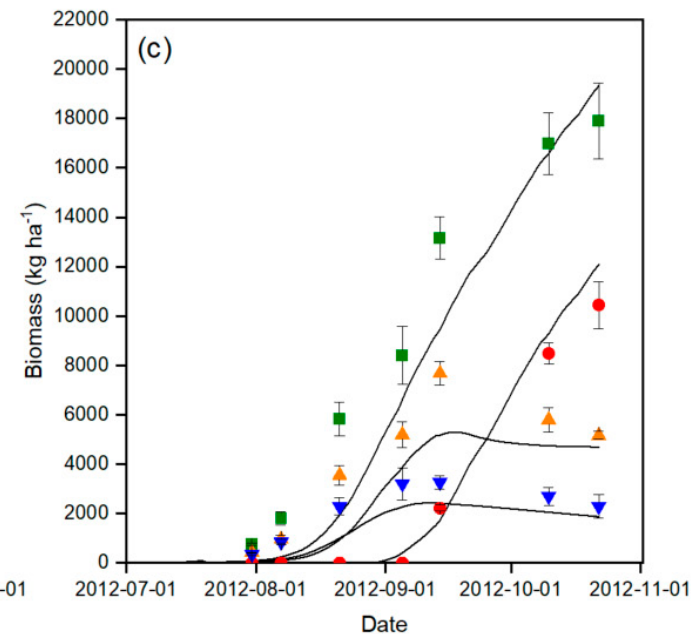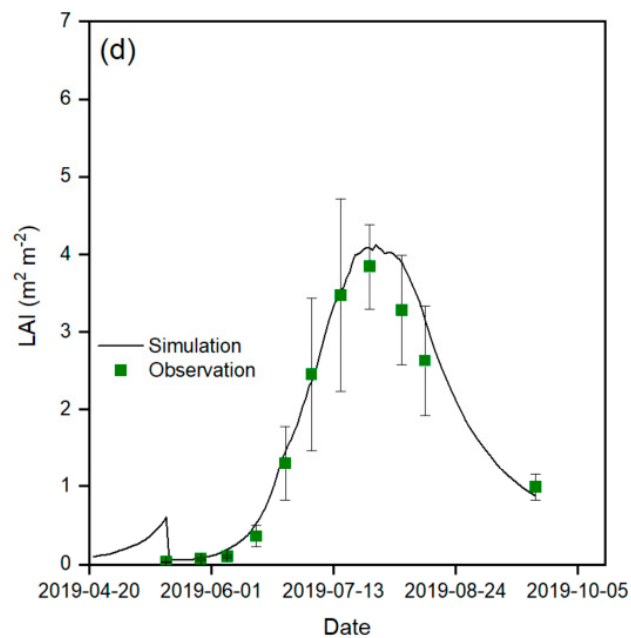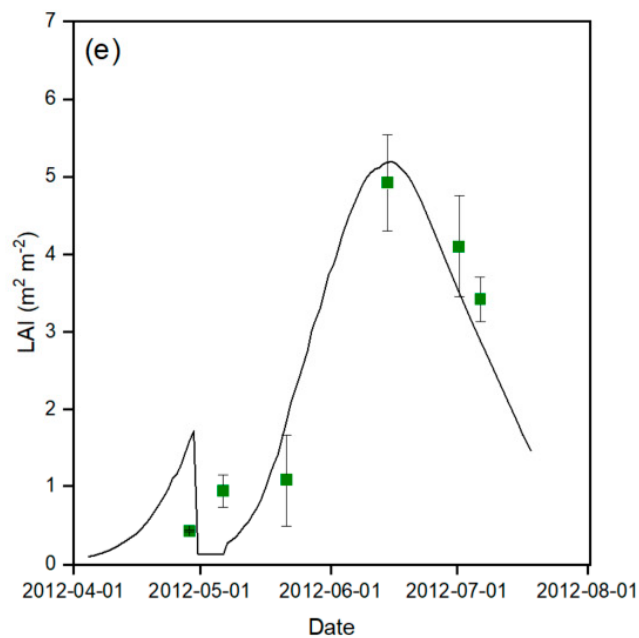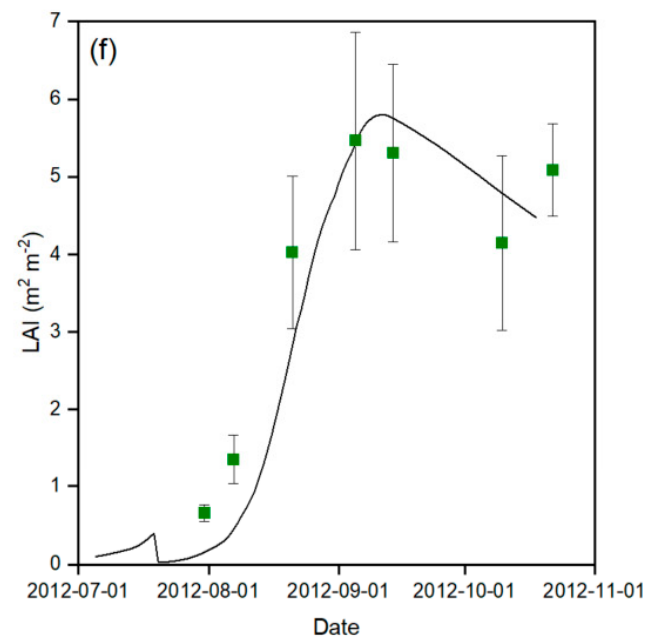

Supplement: Supplementary file 1 [file plants-13-00262-s001.zip › plants-2737594-supplementary.pdf]
